# A man stitches his mouth in the context of a personality disorder

**DOI:** 10.1192/j.eurpsy.2024.1358

**Published:** 2024-08-27

**Authors:** A. Monllor Lazarraga, P. Marques Cabezas, L. Rojas Vazquez, M. Rios Vaquero, G. Lorenzo Chapatte, T. Jimenez Aparicio, C. De Andres Lobo, C. Vallecillo Adame, M. J. Mateos Sexmero, N. Navarro Barriga, B. Rodriguez Rodriguez, M. Fernandez Lozano, M. A. Andreo Vidal, M. Calvo Valcarcel, M. P. Pando Fernandez, P. Martinez Gimeno, G. Guerra Valera

**Affiliations:** ^1^Psiquiatria; ^2^SACYL-Hospital Clínico Universitario Valladolid, Valladolid, Spain

## Abstract

**Introduction:**

A 28 year old patient will be presented. This paramilitary man was brought to the Emergency Room due to an autolytic attempt with Benzodiazepines, along with a mouth suture, in the context of a soon to be resolved problematic ankle osteosynthesis procedure. The patient claimed to be suffering pain, furthermore struggling due to the fact he could not be working due to his ankle issue. Language barrier was a problem during the interview.

**Objectives:**

The objetives of this case is to try to explain the issues that may arise in patients with personality disorders in the context of an autolytic attempt

**Methods:**

This patient will be presented, along with systematic bibliography review of the topic.

**Results:**

The following results were extracted upon the attention given to this patient which was admitted to the Psychiatric Unit.

First of all, the mouth stitches were removed, along with a petition for toxicological analysis. The results gave positive for cannabis and benzodiazepines. The patient was also brought previously this year with another autolytic attempt, this time on cocaine consumption too. Furthermore, a thorough review was made of the other autolytic attempts, including those which happened in his country of origin. The patient has hundreds of small cuts among his arms, from previews cuts made in the past. Furthermore, subcutaneous wounds were auto inflicted in the ER, with a small blade.

Among the whole interview, it was clear he had a personality disorder, with high impulsivity levels and lack of control once the situation overflows.

We also tried to understand the outcome of suturing his mouth. The patient referred his acts of impulsiveness due to his overwhelming situation of both having no job at this moment and the pain he was suffering due to his ankle procedure.

The patient was admitted to our Unit due to the high risk he could repeat this act. Upon arrival, the same day he was admitted, the patient asked if he had to stay at the unit. When explaining the following already told event, furthermore insisting in the possibility of been evaluated by the Traumatology team, he proceeded to try and hang himself with his medical-hospital clothing.

The patient was treated with antipsychotics. Along with Lormetazepam at night. At the end of the hospitalization, and after been evaluated by the Psychiatrist of this Unit, the patient was also treated with Lithium due to its effectiveness in the treatment of autolytic attempts.

**Conclusions:**

Personality disorders are one of the psychiatric pathologies that prevail with greater frequency in autolytic attempts ^1^. Additionally, it should be taken into account the possible ongoing consumption of psychoactive drugs that could also derive in psychopathological decompensation. On top of the following, the use of antipsychotic treatment is indicated for the managing of conduction altercations ^2^, besides Lithium being a great option in managing suicidal temptations ^3^.

**Disclosure of Interest:**

None Declared

